# A Technique to Track Scatterers for Continuous High-speed Plane-wave Ultrasound Simulations based on a Fluid Domain Model

**DOI:** 10.1109/ojuffc.2025.3618637

**Published:** 2025-10-07

**Authors:** Jeffrey A. Ketterling, Geraldi Wahyulaksana, Marisa S. Bazzi, Hadi Wiputra

**Affiliations:** 1 Department of Radiology, Weill Cornell Medicine, New York, NY 10022 USA; 2 Department of Pediatrics, School of Medicine, Stanford University, Palo Alto, CA 94305 USA; 3 Department of Biomedical Engineering, University of Minnesota, Minneapolis, MN 55455 USA

**Keywords:** ultrasound, flow simulation, plane wave, Doppler, vector flow, cardiac

## Abstract

Ultrasound simulations of blood flow are useful to evaluate or optimize new transmit schemes, transducer geometries, or post processing methods such as vector flow. In cases of complex flow, a flow domain model (FDM) is often used to define the time history of the velocity field. Scatterers representing blood cells are seeded in the flow field and their positions are updated each time step after spatial and temporal interpolation of the FDM velocity field. At each time step, the scatterers are passed to an ultrasound simulator to generate synthetic ultrasound backscatter data. Here, a technique is described to continuously track, without temporal discontinuities, a stable concentration of scatterers representing complex flow with reverse, rotational, out-of-plane and/or helical features. The unique aspects of the tracking approach are 1) refresh zones at the input and output flow ports that randomly reseed scatterers each time step, 2) a stagnation threshold to remove low velocity orphaned scatterers near the boundary of the flow field, and 3) continuous tracking of particles in the full flow volume. The method can be adapted to any FDM, ultrasound simulator, transducer, or transmission scheme. To demonstrate the overall pipeline, we use the results of a prior fluid structure interaction (FSI) model of a mouse aorta to generate a continuous high-speed, plane-wave ultrasound simulation over 4 cardiac cycles with a 15-MHz linear array. The data were processed to produce vector flow to validate that the ultrasound vector-flow field was consistent with the FSI velocity field.

## I Introduction

ULTRASOUND simulations are used extensively to evaluate new transmit/beamforming schemes, design transducers, and validate experimental measurements. Simulations have three distinct components: the scattering medium, the ultrasound simulator and the post-processing. A single point can be used to evaluate a transducer point spread function (PSF) or thousands of points can be used to simulate tissue. The concentration of particles is wavelength, λ, and transducer dependent with at least 10 scatterers per unit resolution cell is considered sufficient to produce fully developed speckle [1] where the resolution cell represents the transducer focal volume.

Simulations of flow require managing the time history of the positions of the particles. This task is straightforward for simple trajectories like parabolic flow in a tube or a rotating disk phantom. Dealing with more complex volume flow over a cardiac cycle, such as in the aorta or left ventricle, presents unique challenges to ensure the scatterer collection (i.e., blood) maintains local concentration. For cases of 3D complex flow with reverse, rotational, out-of-plane and/or helical features, a flow domain model (FDM) (e.g., computational fluid dynamic (CFD) or fluid-structure interaction (FSI)) is often employed. FDMs provide many output options including pressure, strain, wall shear stress and velocity, often on a computational mesh grid that varies in time and space.

Linking an FDM to an ultrasound simulation requires an approach to manage the time history of a large collection of randomly seeded scatterers that represent blood. FDMs generally do not track the flow lines of a large concentration of particles but simulations can be performed that account for inertia of particles in the flow or massless particles can be seeded post-simulation. The ultrasound time step is typically 10–100x smaller than the FDM output time step (e.g. 0.05 vs. 5 ms). Thus, temporal and spatial interpolation is necessary to shift the scatterer particles based on the FDM output. The 3D nature of the flow and the need to allow particles to enter or leave the flow domain without impacting local concentration or geometry present technical challenges to generate a continuous movement of scatterers over many cardiac cycles.

The basic process of tracking scatterers using and FDM and simulating ultrasound has been described in the literature. Swillens et al. [2] simulated the flow field in a rigid carotid artery bifurcation [3] and a distensible artery and used interpolation to determine scatterer shift each time step based on FDM velocity fields. However, the scatterers were reset after the desired Doppler ensemble length to avoid “an artificial dilution and aggregation of point scatterers”. The authors also used the particle reset approach with a mouse aorta model [4]. Sun et al. [5] demonstrated a pipeline starting with a CFD model of left ventricle flow and ending with color Doppler but the simulation did not include out-of-plane flow, the scatterers were restricted to a single plane, but the authors did not state how often scatterers were reseeded. Van Cauwenberge et al. [6] used a multiphysics package to model blood flow in simplified pediatric left ventricle but also with the scatterers periodically reset. Balocco et al. [7] used a finite element package (COMSOL, Inc., Burlington, MA) to model the flow in a carotid and then Doppler estimates were found line by line with particle position reset every 10 transmits.

While scatterer reset approaches are sufficient for simulating acquisitions that are line based and/or ensemble based, they introduce discontinuities in the slow-time/speckle and are not able to replicate the data from continuous high-speed plane-wave acquisitions over multiple cardiac cycles. Avdal et al. [8] proposed a method to combine particle tracking and a flow simulator by pre-calculating a transducer’s PSF along defined flow lines. However, the approach required recalculation anytime the transducer properties changed, and the authors noted that their approach was not appropriate for complex pulsatile flow.

Here, we present a technique to track a collection of scatterers in a complex, pulsatile flow that is not tied to a particular FDM model, transducer or ultrasound simulator and does not require periodic particle reseeding to keep local concentration stable. The ability to replicate *in vivo* flow without any discontinuities in the slow time allows transmit schemes and processing methods to be evaluated under realistic conditions. To demonstrate the processing pipeline, we employ a previously derived FSI simulation of flow in a mouse aorta. We specifically seek to address the challenging case of particle tracking over an arbitrary number of cardiac cycles for continuous high-speed plane-wave acquisitions. The impact of design parameters such as particle concentration, refresh zones and a stagnation threshold are discussed in the context of the example case. Finally, we validate the full simulation pipeline by generating vector flow and comparing back to the FSI velocity field.

## II Methods

We are mainly focused on managing the movement of scatterers in a complex flow that provide the link between any FDM and an ultrasound simulator. However, the ability to generate an ultrasound simulation from the scatterer collection is the ultimate purpose of our approach. Therefore, we will use an exemplary mouse aorta FDM to demonstrate the steps of the process of going from an FDM, to managing the scatterer collection, to an ultrasound simulation of high-speed plane-wave imaging. The key steps of the process are preparing the FDM for interpolation, defining tuning parameters, managing the translation of the scatterers, and producing the final ultrasound output that is then processed for vector flow.

### A. Preparing FDM Output

The first step of generating the scatterer model is deriving an FDM time history which generally will span a full cardiac cycle. The key outputs required from the FDM for particle tracking are a mesh grid and the velocity at each mesh grid point over a series of time steps. If the FDM output represents a non-uniform mesh grid $\left(x_{f},y_{f},z_{f}\right)$ an interpolation operation 𝓡 is useful to align the FDM velocity field, $U_{f}$, onto a uniform grid, $\left(x_{g},y_{g},z_{g}\right)$, with spacing d such that

(1) $$V_{g}^{m}=𝓡\left(x_{f},y_{f},z_{f},U_{f}^{m},x_{g},y_{g},z_{g}\right)$$

where $V_{g}$ is velocity on the new grid and the interpolation is repeated for each of the m time steps of the FDM time series $t_{f}$.

The FDM model may include just points in the fluid domain. Therefore, before the above interpolation, a set of null velocity points outside the flow region should be appended to the FDM to avoid potential interpolation errors near the edge of the flow. We defined the null points to be any point on the new interpolation grid that was $>\sqrt{3}*d/2$ from the nearest point in the dense collection of FDM flow points. The value $\sqrt{3}*d/2$ represents the midpoint between opposite corners of a cube of length d. The indexes of the uniform grid points inside and outside the flow domain were stored for later use.

### B. FDM of Mouse Aorta

**FIGURE 1.**
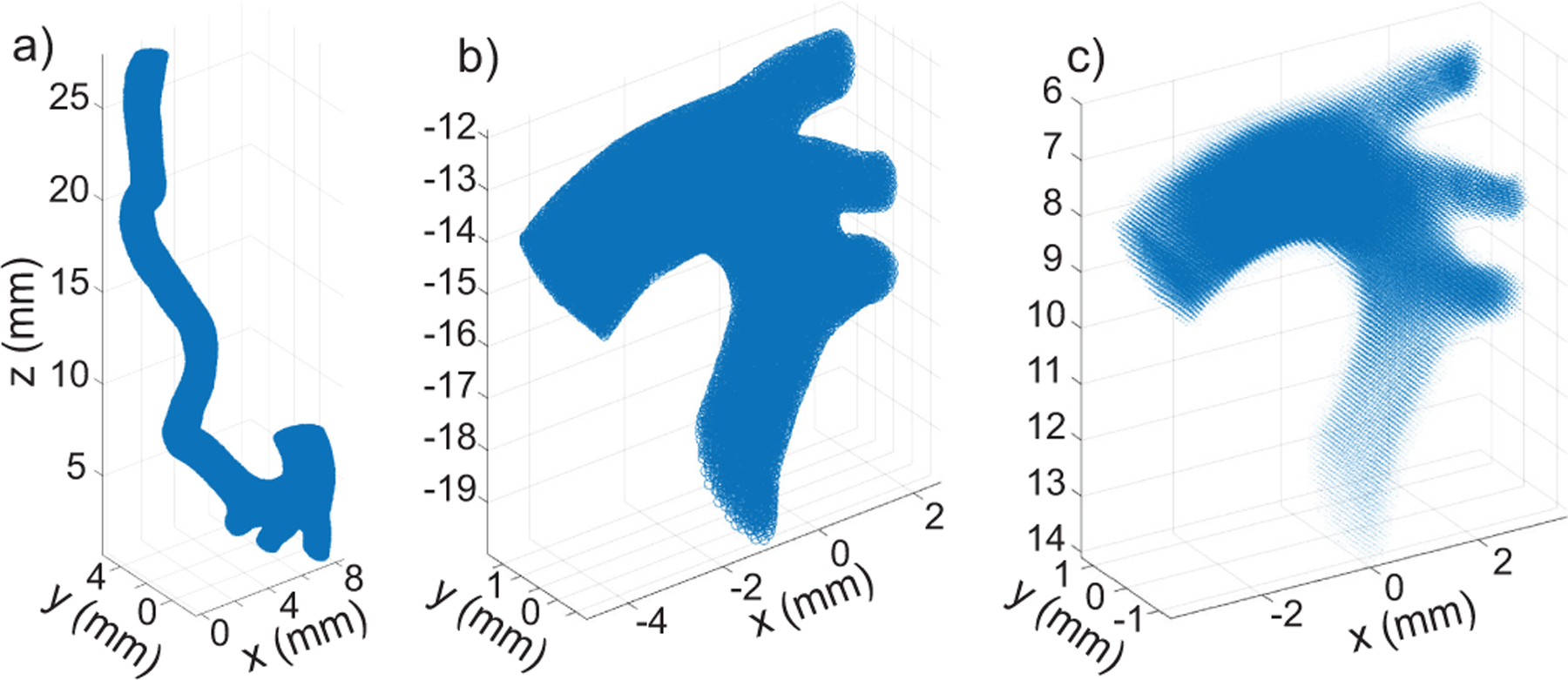
a) Example of fluid domain of a mouse aorta FSI simulation. b) The FSI’s fluid domain is rotated and cropped into a standard view for mouse imaging with the ascending aorta to the left, the descending aorta flowing downward, and the aortic branches towards upper right. c) Interpolation of FSI onto a d=0.1mm grid.

We demonstrate the process of preparing the FDM using a previously published FSI simulation of a mouse aorta [9]. Briefly, the *in vivo* aortic geometry was obtained with magnetic resonance imaging (MRI) [Figure 1]. The fluid domain inlet and outlet boundary conditions were obtained from [10]. Inlet boundary conditions were set as a Dirichlet boundary, based on temporally varying flow rate obtained from reduced order model of the heart [10] (obtained flow rate is illustrated as Figure 2b in [9]). The outlets were set as Resistor-Capacitor-Resistor (RCR) boundaries. The values of the resistors and capacitors of the RCR boundaries were calibrated to match the pressure waveform obtained from a catheter measurement in [10] and the flow distribution in Table 6 of [10].

For the solid domain, inlets and outlets have fixed displacement boundaries. The outer wall was assigned with a Robin type boundary to model the surrounding tissue’s support following Moireau et al. [11]. The aorta wall was modeled as a Holzapfel, Gasser, and Ogden material [12] with parameters fitted to the *ex vivo* biaxial stretch-inflation mechanical test of the excised aorta [9]. Two-way solid and fluid domain FSI were performed using the open-source software SvFSI [13] until the normalized residual of the stress-equilibrium equations of the solid domain and the Navier-Stokes equations of the fluid domain was less than 10^−4^.

The blood was modeled as a non-Newtonian fluid using the shear-thinning Carreau-Yasuda model (2) where the apparent viscosity $\left(\eta \right)$ was a function of infinite-shear-rate viscosity $\left(\eta_{\infty }\right)$, zero-shear-rate viscosity $\left(\eta_{0}\right)$, shear rate $\left(\dot{\gamma }\right)$ and several power law constants $\left(n,a,\text{λ}\right)$. The full description of the viscous model and input parameters are described in [9] and parameter values are summarized in Table 1.


(2) $$\eta \left(\dot{\gamma }\right)=\eta_{\infty }+\left(\eta_{0}-\eta_{\infty }\right){\left[1+{\left(\text{λ}\dot{\gamma }\right)}^{a}\right]}^{\frac{n-1}{a}}$$

The FSI output used for the particle tracking represented the 10^th^ cardiac cycle. The full extent of the FSI aorta model geometry did not correspond to what would be visible with ultrasound *in vivo* [Figure1(a)]. Therefore, we cropped and rotated the data into an appropriate orientation for an *in vivo* ultrasound image [Figure 1(b)] and then interpolated onto a uniform grid with d=0.1mm spacing that, after interpolation resulted in 27,937 grid points in the flow field [Figure 1(c)].

### C. Define Refresh Zones

The input forcing function and boundary conditions of an FDM define the flow field. Translating a collection of scatterers over several cardiac cycles presents some challenges because scatterers need to enter or leave the simulation space. One way to manage this is very long input and output tracks but that adds burden to the FDM simulation in terms of more points on the grid, shifts the forcing function upstream and the length will need to change based on the ultrasound simulation requirements.

**FIGURE 2.**
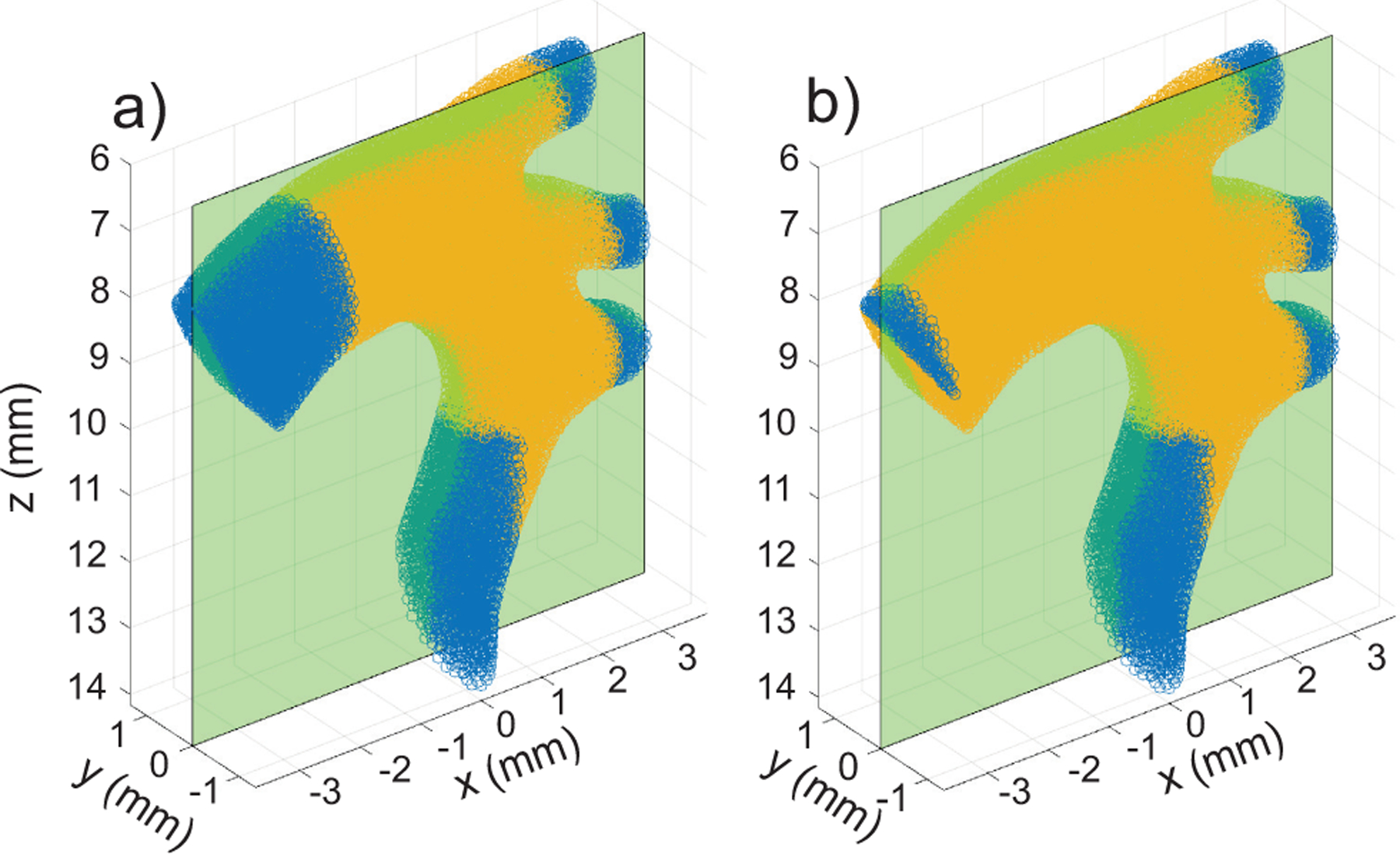
Mouse aorta model with refresh zones marked in blue and the scatterer space used by ultrasound simulator in yellow. Two examples are provided: a) refresh zones sufficiently large and b) the ascending aorta zone too small. The reference plane through y=0 represents the image plane of ultrasound.

Our approach is to define source/sink zones at the entrance and exit locations of flow. The points in the zones are not passed to the ultrasound simulator and the zones are refreshed with randomly generated scatterers before each simulation time step to ensure that new scatterers are always available to enter the simulation, and old scatterers can exit. This approach maintains slow-time temporal correlation and simulates continuous *in vivo* data acquisition. If a zone is too small, null space with no scatterers will develop and propagate. For our aorta example, the refresh zones are indicated in blue in Figure 2(a).

### D. Stagnation Threshold

Local concentration may be impacted when particles accumulate because they are directed into a zone with near zero velocity or particles exit the valid flow volume and end up stranded. However, we want to avoid removing points that may have momentarily stopped during pulsatile flow.

We introduce a stagnation threshold to remove low velocity points at the edge of the flow region. Removal of stagnant points is based on two threshold parameters. First, after each time step, all points below a defined velocity are identified. Second, the distances between these points and the nearest non-flow grid point are found. If a flow point is below the velocity threshold and below a defined distance threshold from the nearest non-flow grid point (e.g., <2*d where d is grid spacing), the flow point is considered stagnant and removed from the simulation. The velocity and distance thresholds are design parameters that need to be tuned such that particle concentration remains steady over time.

### E. Ultrasound Simulator and Post-processing

We used the native Verasonics (Kirkland, WA) simulation mode with a 15-MHz linear array (L22–14vX, Verasonics) to generate the ultrasound data of flow in the mouse aorta. The resolution cell representing the focal volume of the linear array used in plane-wave excitation was defined as 0.4 mm x 0.53 mm x 0.2 mm or 4λ×5.3λ×2λ, lateral, elevation, and axial, respectively. To maintain > 10 scatterers per resolution cell [1], a concentration of $>\;{0.23\;\text{pts/λ}}^{3}$ is necessary $\left(\lambda \;=\;0.1\;\text{mm}\;\text{at}\;15\;\text{MHz}\right)$. We did not include a background tissue in the simulation because clutter filtering is normally used to remove the static tissue signal component.

To generate continuous ultrasound data, we used a high-speed plane wave transmissions with 3 transmit angles [−10°,0°,10°]. The absolute pulse repetition frequency (PRF) was 30 kHz which yielded a 10-kHz effective frame rate after compounding the three transmit angles. We used our own MATLAB (The MathWorks Inc., Natick, MA) code to beamform the in-phase and quadrature signal (IQ) using a constant F-number=2 with a 25 *µ*m x 25 *µ*m pixel spacing, and the simulation spanned 4 cardiac cycles (400 ms).

While we tracked the full volume of particles each time step, only the points $\left|y\right|<0.25\;\text{mm}$ were passed to the ultrasound simulator. This reduced the calculation time by removing scatterers outside the elevation slice that contribute little to the backscatter. For example, an aorta simulation starting with a $\text{0}{\text{.23}\;\text{pts/λ}}^{3}$ concentration tracks ≈ 6700 scatterers but only ≈ 2100 are included in the ultrasound simulation each time step.

Color Doppler was obtained from the IQ data after compounding and applying a high-pass filter along the slow-time axis with a normalized 0.1 cut off. Lag-one autocorrelation [14] was used for the Doppler phase shift estimates over a 64-point sliding window with a 4-point shift. Doppler spectrograms were calculated with the MATLAB function *spectrogram* using a 64-point sliding window, zero padded to 256 points and a 4-point shift. The Doppler aliasing velocity for the 15-MHz transducer and 10-kHz PRF was 25.5 cm/s. Finally, we calculated vector-flow estimates using methods described previously [15]–[17]. Briefly, a multi-angle plane-wave, Doppler estimation strategy was used with a least-squares fitting process to estimate the $V_{x}$ (lateral) and $V_{z}$ (axial) velocity components at all pixel locations. To minimize artifacts in the vector estimates during slow flow, a power threshold was applied to the high-pass filtered IQ. Lag-one estimates were forced to zero when the IQ power was below the threshold.

### F. Summary of Processing Steps

The sequence of steps to produce an ultrasound simulation from an FDM using our scatterer tracking technique are:

1. Within the box defined by the $\left(x_{g},y_{g},z_{g}\right)$ spatial limits of the flow region, a collection of scatterers are generated. A Gaussian weighted scattering amplitude $\left(A\right)$ is also included for each scatterer to produce the set of points $\left(x_{s},y_{s},z_{s},A_{s}\right)$
2. The seeded scatter points outside the flow region, where flow was defined as zero, are removed.
3. The velocity of each scatterer is found via spatial and temporal interpolation from the stack of interpolated FDM data
(3) $${\bar{V}}_{g}^{t_{n}}=𝓡\left(x_{g},y_{g},z_{g},t_{f},V_{s}^{m},x_{s},y_{s},z_{s},t_{n}\right)$$
  where ${\bar{V}}_{s}^{t_{n}}$ is the interpolated velocity vector of each scatterer at the time $t_{n}$. If the simulation time point exceeds the FDM total time, the simulation time is rolled over to the start of the FDM time scale.
4. The scatterers are displaced to their new position based on the time step $\Delta t=t_{n+1}-t_{n}$
(4) $$\left(x_{s}^{n+1},y_{s}^{n+1},z_{s}^{n+1}\right)=\left(x_{s}^{n},y_{s}^{n},z_{s}^{n}\right)+{\bar{V}}_{s}^{t_{n}}*\Delta t.$$
5. All points in the refresh zones are removed from the scatterer collection.
6. Any scatterers that are below the velocity and edge-of-flow distance thresholds are considered stagnant and removed.
7. The scatterers within the defined elevation slice are passed to the ultrasound simulator.
8. The refresh zones are randomly repopulated and appended to the scatterer collection.
9. Return to step 3 until last frame of ultrasound simulation.
10. Post-processing (beamforming, Doppler, speckle tracking, etc.).

## III. Results

### A. Scatterer Trajectories in Complex Flow

**FIGURE 3.**
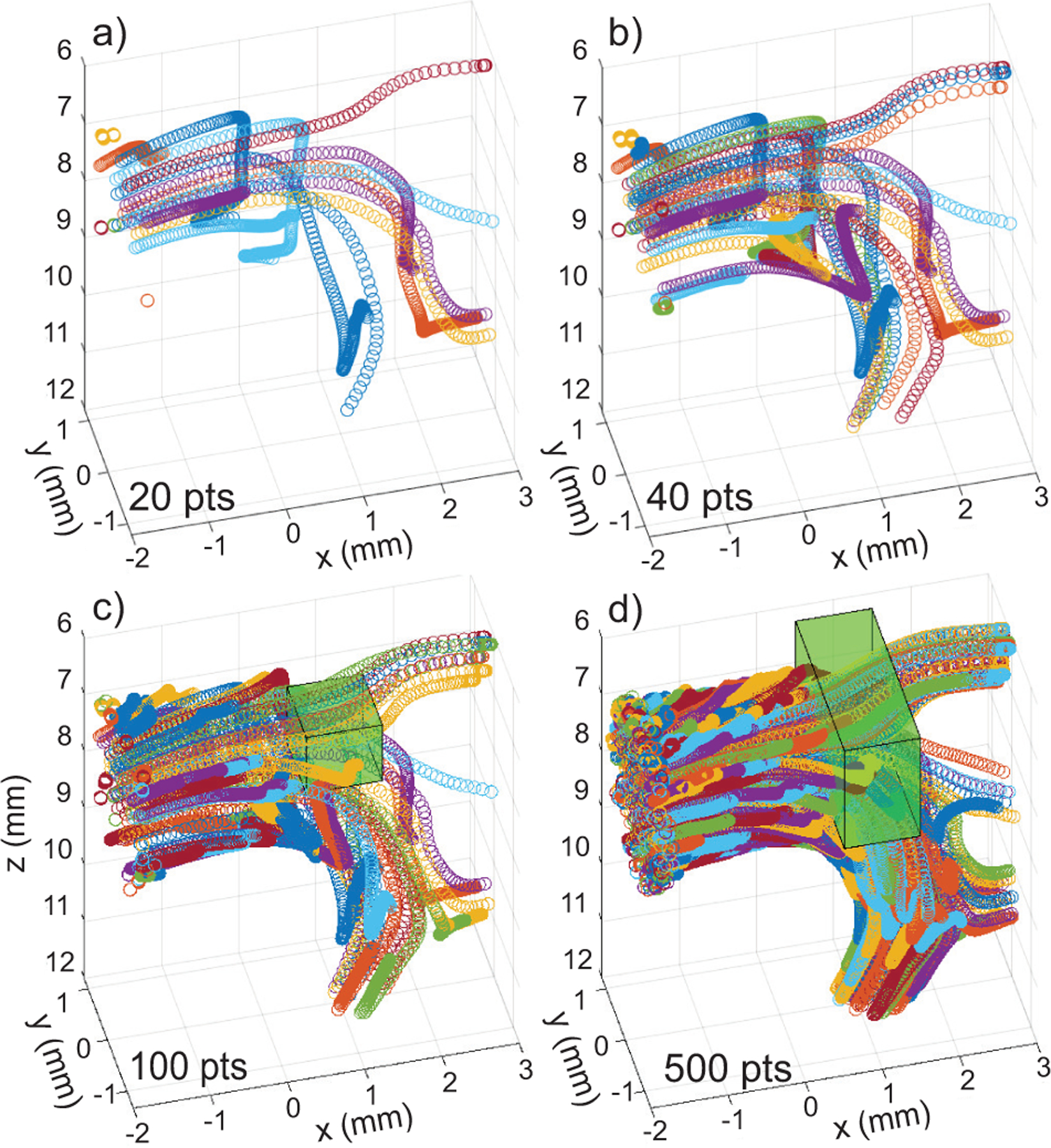
Trajectories of scatterers over one cardiac cycle for cases of a) 20, b) 40, c) 100, and d) 500 scatterers. The scatterers were randomly seeded at the entrance of the ascending aorta model. Each particle is assigned a color. Particles in the center of the flow were most likely to exit the simulation flow field. As the particle count increased, the mapping of the flow region became more defined. The green volumes (c and d) are particle tracking volumes.

Complex pulsatile *in vivo* flow may have a reverse, rotational and/or helical trajectory. Figure 3 provides examples of our FSI flow paths in the mouse aorta over the span of 1 cardiac cycle when seeding a small collection of points at the ascending aorta entry. Four cases are shown for increasing number of initial scatterers: 20, 40, 100 and 500. The 20-point case revealed the complicated trajectories where the total distance traveled of each scatter over 100 ms was not equal and some scatterers reversed in direction or traveled in a helical forward flow path.

The scatters in the center of the flow traveled the furthest with some reaching the descending aorta and some diverting into the carotid branches. The particles near the edge of the flow did not exit the flow region during one cardiac cycle. Some particles moved very little while others had a significant shift in y position. When viewing the path of 40 initial scatterers, the flow region was further emphasized, and the overall pathways became more tangled. Increasing further to 100 and 500 points started to reveal the carotid branches and the full flow region became apparent. The trajectory tracking also confirmed that the simulation restricted the particles to the flow region.

### B. Stability of Particle Concentration

**FIGURE 4.**
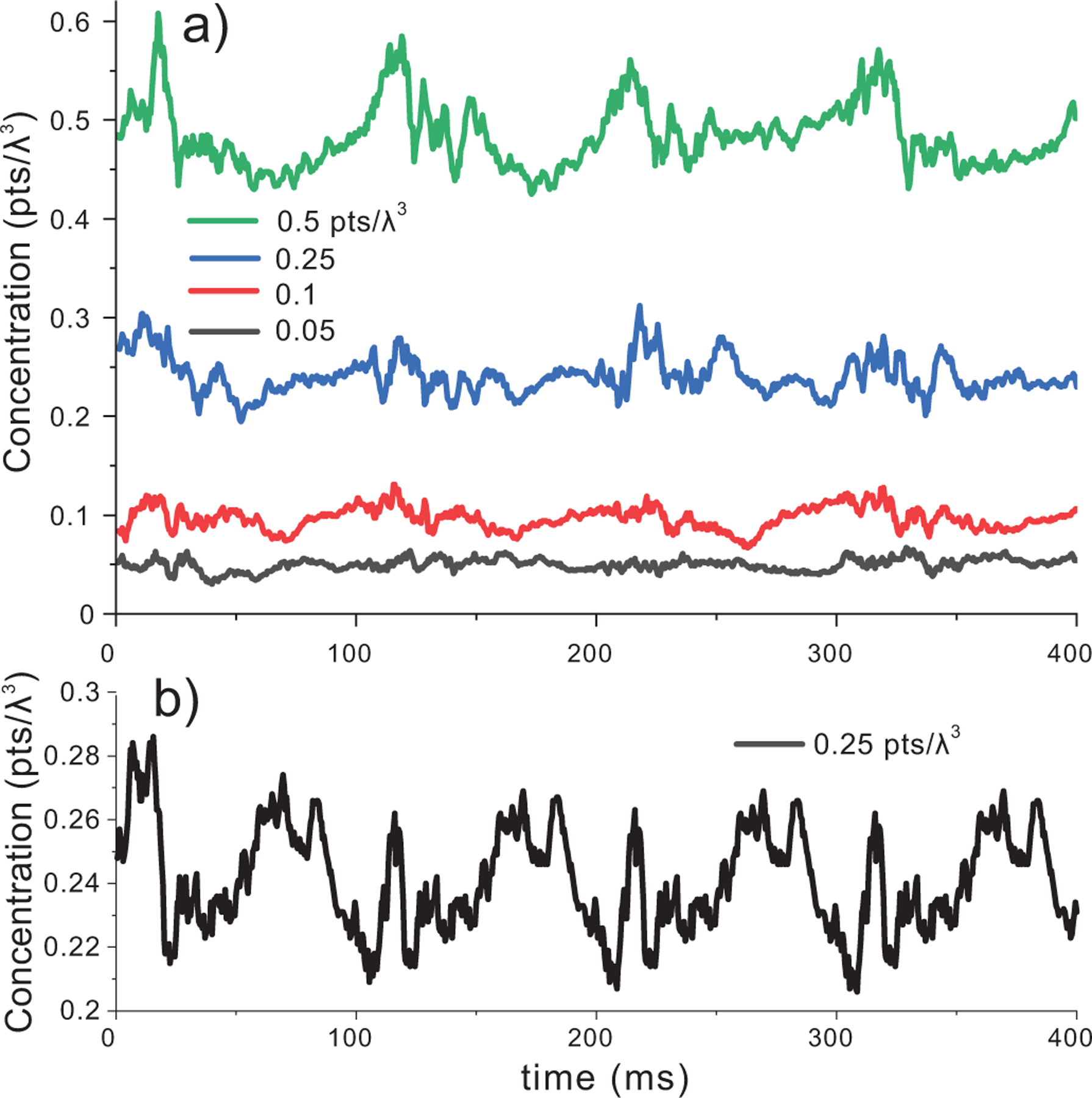
a) Scatterer concentration as a function of seeding concentration within a 10×10×10 ${\text{λ}}^{3}$ (marked in Figure 3(c)). The result spans 4 cardiac cycles using a 0.5-ms time step. b) If the refresh zones were non-randomly reseeded each time step, the time history repeats each cardiac cycle after an initial transient.

A key aspect of particle tracking is to ensure the local concentration stays relatively uniform. We evaluated local concentration over 4 cardiac cycles with a fully populated collection of points in the aorta for initial concentrations of $0.05,\;0.1,\;0.25\;\text{and}\;0{.5\;\text{pts/λ}}^{3}$. These concentrations represent 2.1, 4.2, 10.6 and 21 scatterers per resolution cell, respectively. The simulations used a 0.5-ms time step (2 kHz, 800 volumes) and local concentration was tracked in a 10×10×10 ${\text{λ}}^{\text{3}}$ volume centered in the middle of the aorta at [1 0.25 7.5] mm such that it did not contain edge flow (marked in Figure 3(c)). The stagnation threshold was set at 1 mm/s with a search distance of 2*d. Figure 4a summarizes the concentration versus time for the four initial seeding concentrations. The mean values of $\text{0}{\text{.05}\;\text{pts/λ}}^{3}$ ±0.006, 0.096 ±0.011, 0.24 ±0.018, 0.48 ±0.032 were consistent with the expected means. If the refresh zones were updated with the same particle collection each time step, the concentration curve repeats each cardiac cycle after the initial random particles were flushed out [Figure 4(b)].

### C. Stagnation Threshold

**FIGURE 5.**
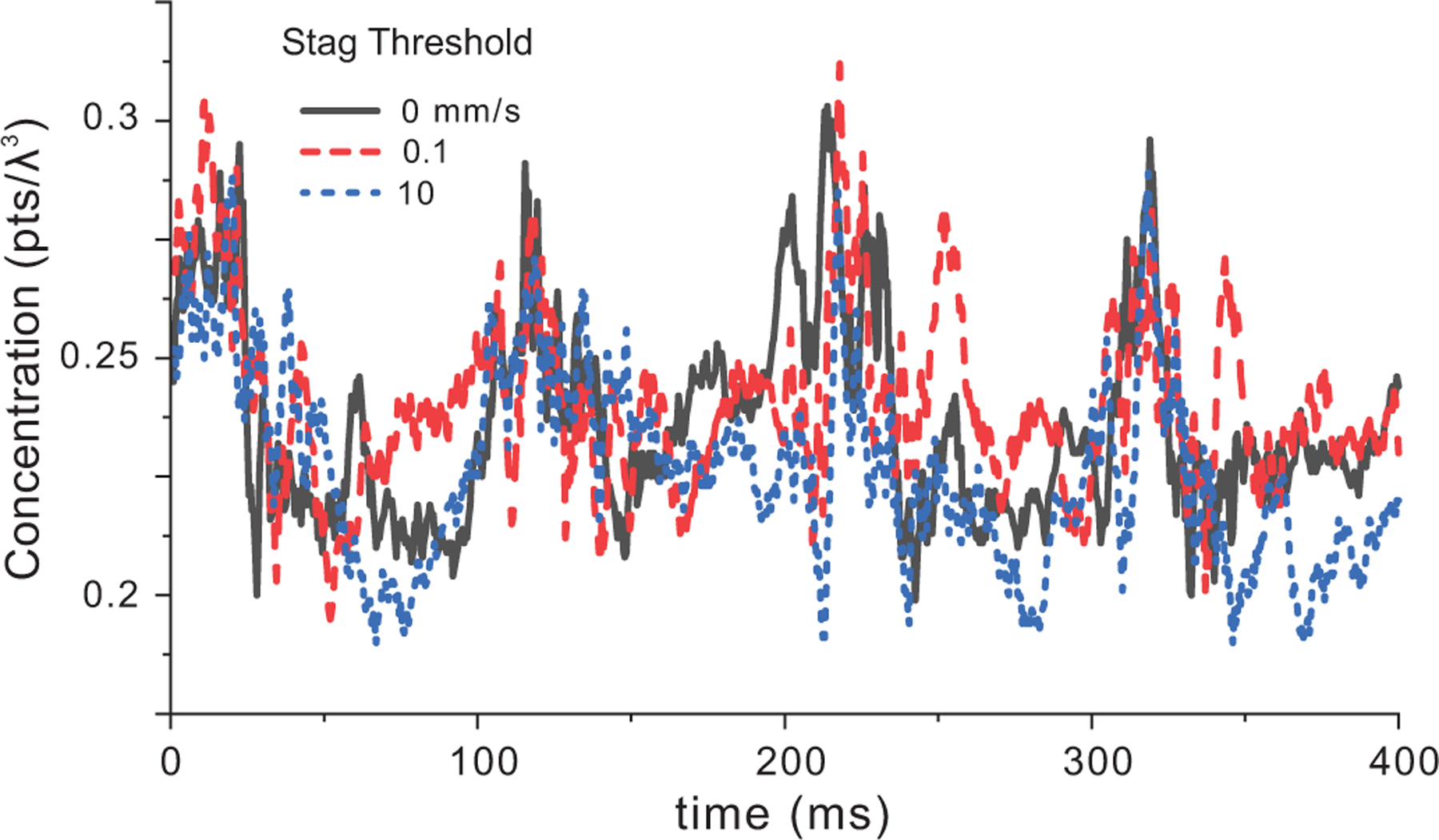
Scatterer concentration as a function of velocity threshold over 4 cardiac cycles in a central 10×10×10 ${\text{λ}}^{3}$ volume (marked in Figure 3(c)) starting with a $0.25\;\text{pts}{\text{/λ}}^{3}$ concentration. The distance threshold was 2*d for all cases.

Figure 5 provides examples of the effect of velocity threshold on concentration within a central 10×10×10 ${\text{λ}}^{3}$ volume [Figure 3(c)]. The simulations were seeded with a $\text{0}{\text{.25}\;\text{pts/λ}}^{3}$ concentration and only points a distance <2*d from edge of the flow were removed. For velocity thresholds of 0, 0.01, and 10 mm/s, the mean concentrations remained stable at $\text{0}{\text{.24}\;\text{pts/λ}}^{3}\pm 0.02$, 0.24±0.018, 0.23±0.02, respectively (expected mean of 0.25).Because the central volume that was not near the flow field edge, the velocity threshold had minimal impact on concentration.

**FIGURE 6.**
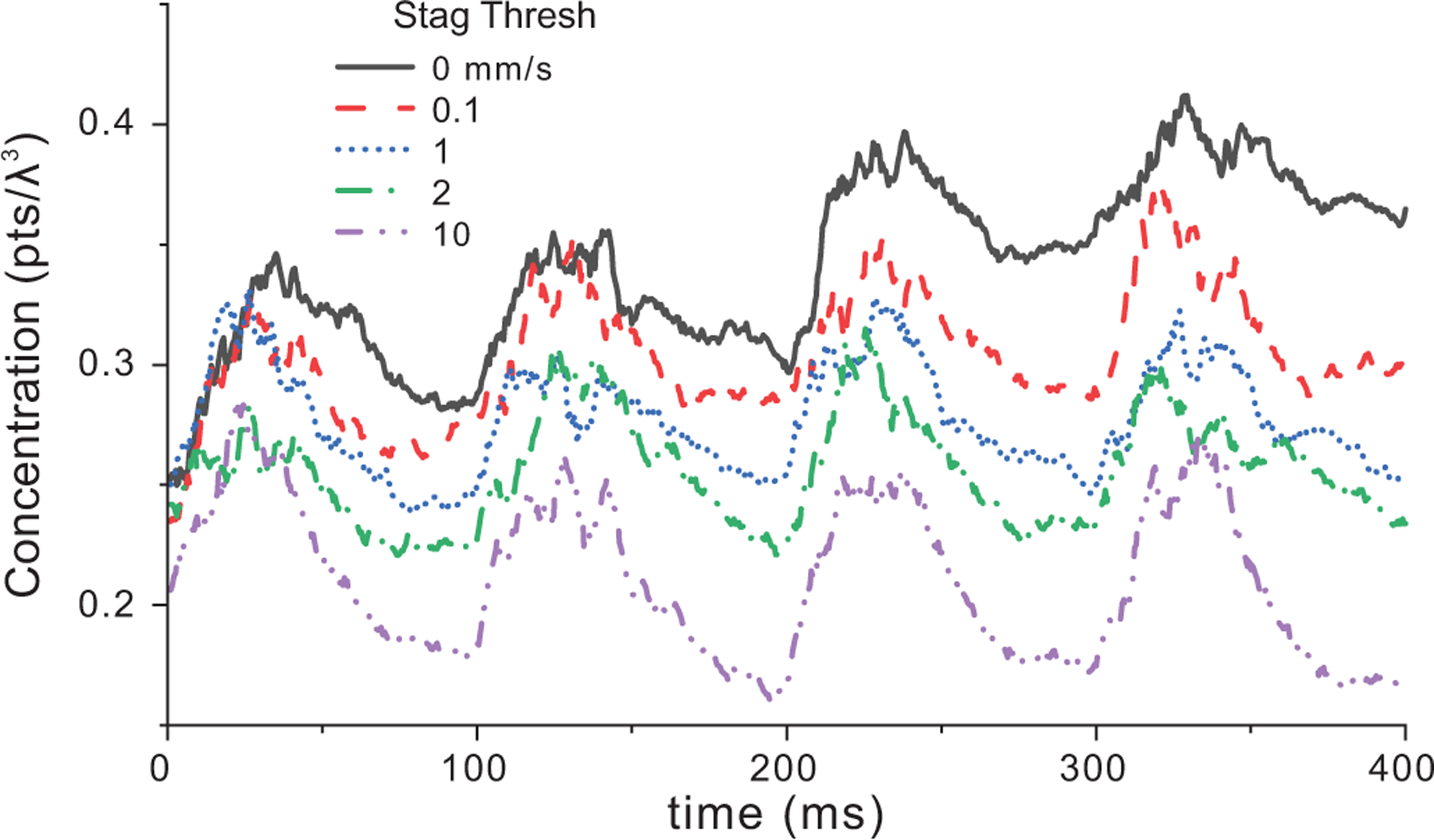
Scatterer concentration as a function of velocity threshold over 4 cardiac cycles normalized to the tracking volume that included the aorta edge marked in Figure 3(d). The starting concentration was $0.25\;pts{\text{/λ}}^{3}$ and the distance threshold for stagnation was 2*d for all cases.

When we maintain the concentration at $\text{0}{\text{.25}\;\text{pts/λ}}^{3}$ and expanded the tracking volume in the ±y and −z -directions to include a portion of the aorta wall (marked in Figure 3(d)), the impact of the velocity threshold at the edge of flow space became apparent [Figure 6]. For velocity thresholds of 0 and 0.1 mm/s, a steady increase in concentration was observed as particles built up [Table 2]. At a threshold of 1 mm/s, the concentration stabilized and at an extreme 10 mm/s a notable overall drop in concentration was observed because an excessive number of particles were removed at the edge of flow.

**FIGURE 7.**
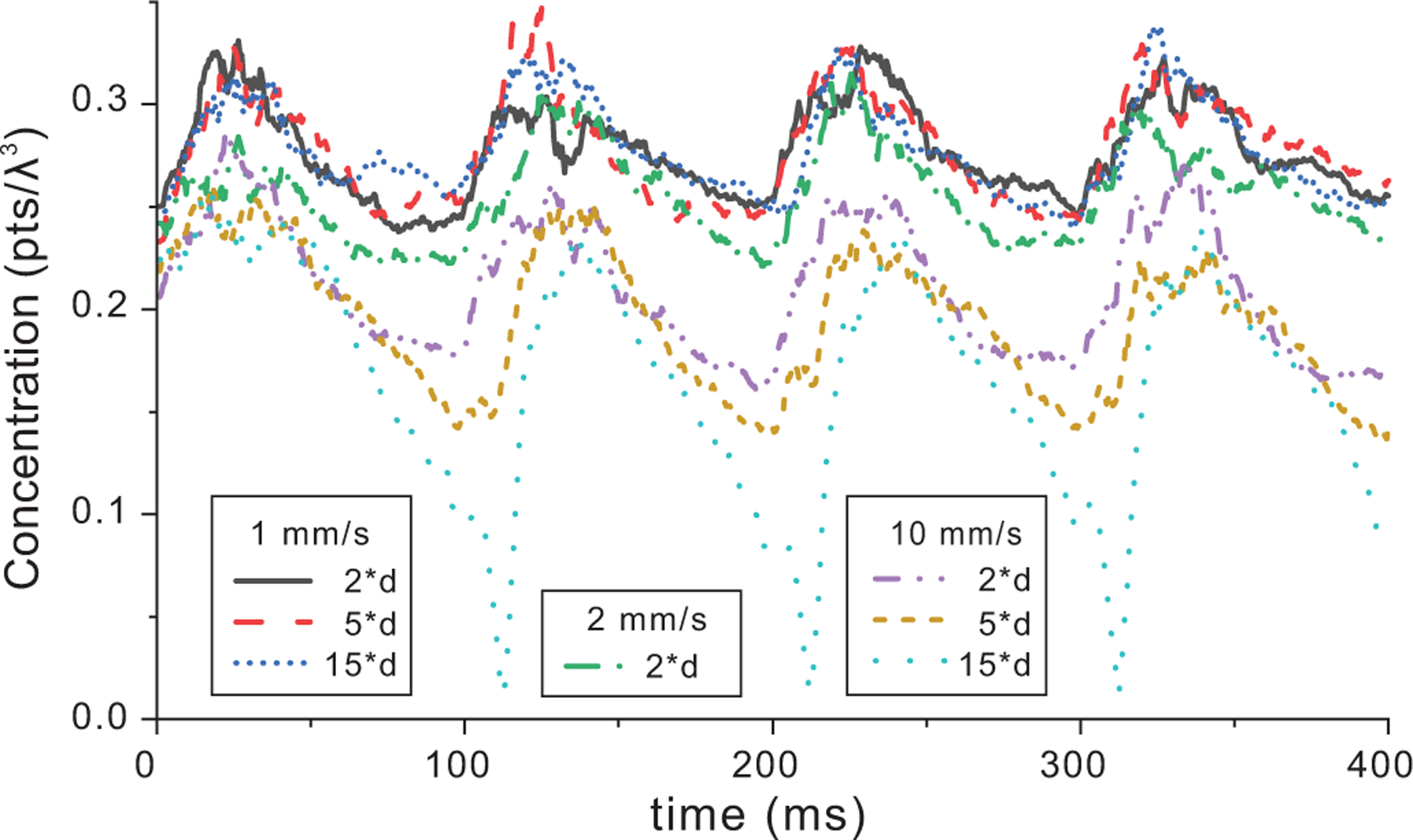
Scatterer concentration as a function of velocity threshold and distance threshold over 4 cardiac cycles normalized to the tracking volume encompassing the aorta edge (marked in Figure 3(d)) starting with a $0.25\;\text{pts}{\text{/λ}}^{\text{3}}$ concentration. The 1 mm/s and 15*d case was unchanged from the 2*d case. The 10 mm/s cases saw more and more points removed each cardiac cycle as distance increases from 2*dto3*dto15*d.

The effect of the distance test for stagnant points at the edge of the flow field is shown in Figure 7 and the trends are summarized in Table 2. When the velocity threshold was 1 mm/s and the distance threshold was increased to 15*d (effectively full volume), the concentration curve was very similar to the 2*d case [Figure 6]. This result implies that any points < 1 mm/s were already confined to the boundary of flow. For a velocity threshold of 10 mm/s, more and more points were removed during the diastole phase each cardiac cycle as the test distance increased from 2*dto3*dto5*d. While 10 mm/s is an extreme velocity threshold that would not be used in practice, the results highlight the importance of restricting the stagnant point search region to the edge of the flow to avoid the potential of removing a substantial number of points in the central flow path.

For our simulations, we chose 1 mm/s and 2*d as the stagnation thresholds because the linear fit slope of the concentration over time was nearly flat (Mean-Int in Table 2) although the mean was somewhat higher then the seeded value of $\text{0}{\text{.25}\;\text{pts/λ}}^{\text{3}}$. Other threshold values would lead to similar results as can be seen in Figure 7 and Table 2 where all the cases of 1 and 2 mm/s result in similar scatterer time histories.

### D. Impact of Refresh Zone

The remaining design parameter is the choice of refresh zones. There must be sufficient volume available in the refill zone to maintain the local concentration in the simulation space after displacing the particles by $V_{s}^{t_{n}}*\Delta t$. The distance each scatterer travels each time step factors into choice of the refresh zone. Here, we provide an example of sufficient and insufficient refresh volumes.

**FIGURE 8.**
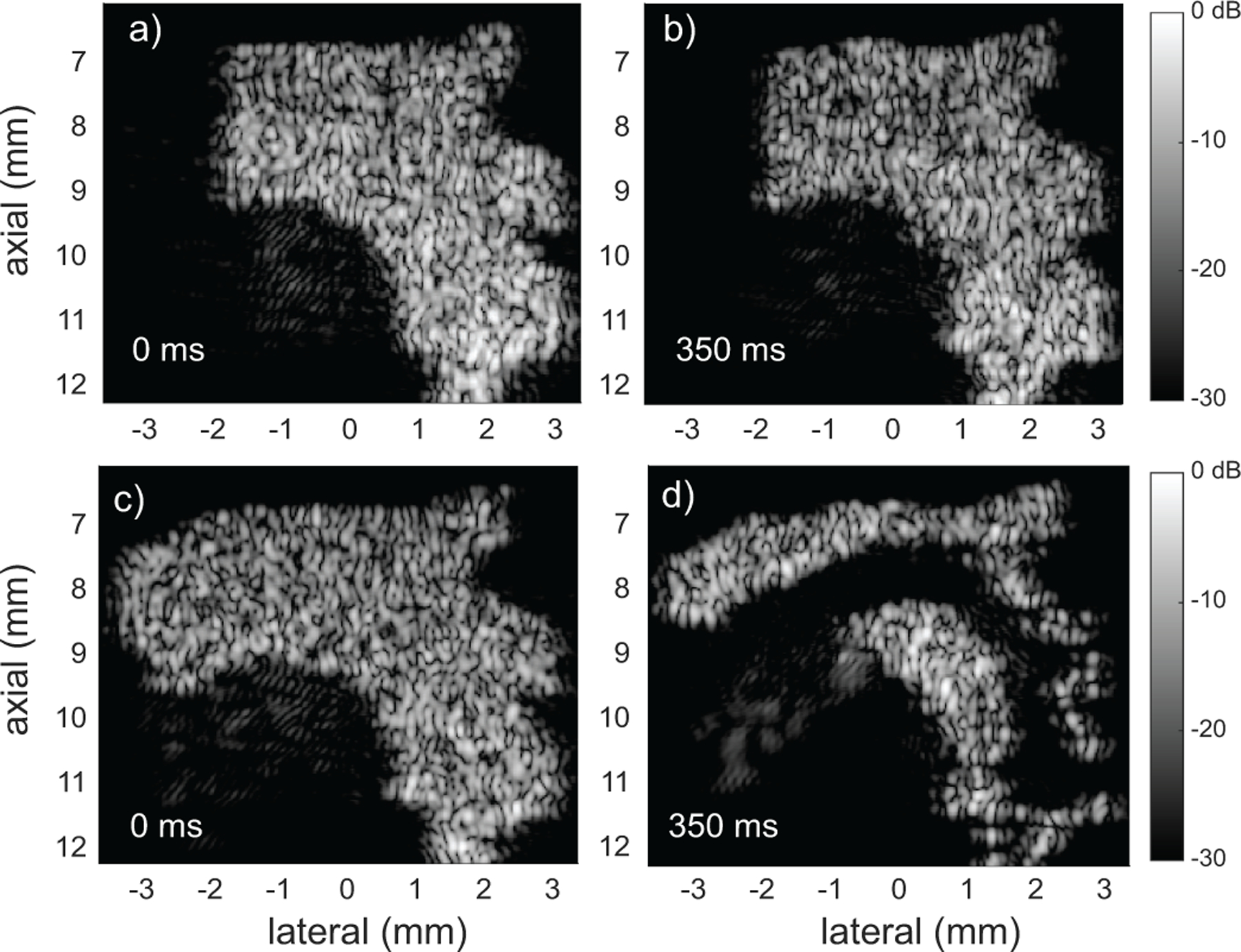
Simulation of flow in mouse aorta over 4 cardiac cycles with sufficient (a, b, and Mm1) and insufficient (c, d, and Mm2) refresh zone volume at the input of ascending aorta. The sufficient case showed no effective change from 0 to 350 ms. The insufficient case was unable to maintain local concentration and the central aorta quickly cleared of scatterers.

We simulated a plane-wave imaging case with the parameters in Sec. IIE using a 1 mm/s velocity threshold, a 2*d distance threshold, $\text{0}{\text{.25}\;\text{pts/λ}}^{\text{3}}$ concentration, three transmit angles, and a 10 kHz effective frame rate after compounding. The refresh zones were first defined as shown in Figure 2(a) and then with the refresh zone volume located at the ascending aorta input drastically reduced [Figure 2(b)]. Figure 8 shows B-mode images at the initial time step and at 350 ms (3.5 cardiac cycles). For the case with sufficient refresh volume, the B-mode images at 0 ms and 350 ms [Figure 8(a) and (b)] showed little difference other than a change in speckle pattern. For the case with insufficient refresh volume, the B-mode images at 350 ms [Figure 8(b)] clearly did not maintain a stable concentration relative to the start time [Figure 8(b)].

### E. Flow Simulations: Doppler and Vector

Once a continuous B-mode sequence of complex flow has been generated we can undertake flow processing using the same approach as would be applied to *in vivo* data. We focus on classic color Doppler, spectrograms and vector flow to further demonstrate the robustness of the full simulation pipeline. We process the data from Figure 8(a) over the 4 cardiac cycles (Mm1).

**FIGURE 9.**
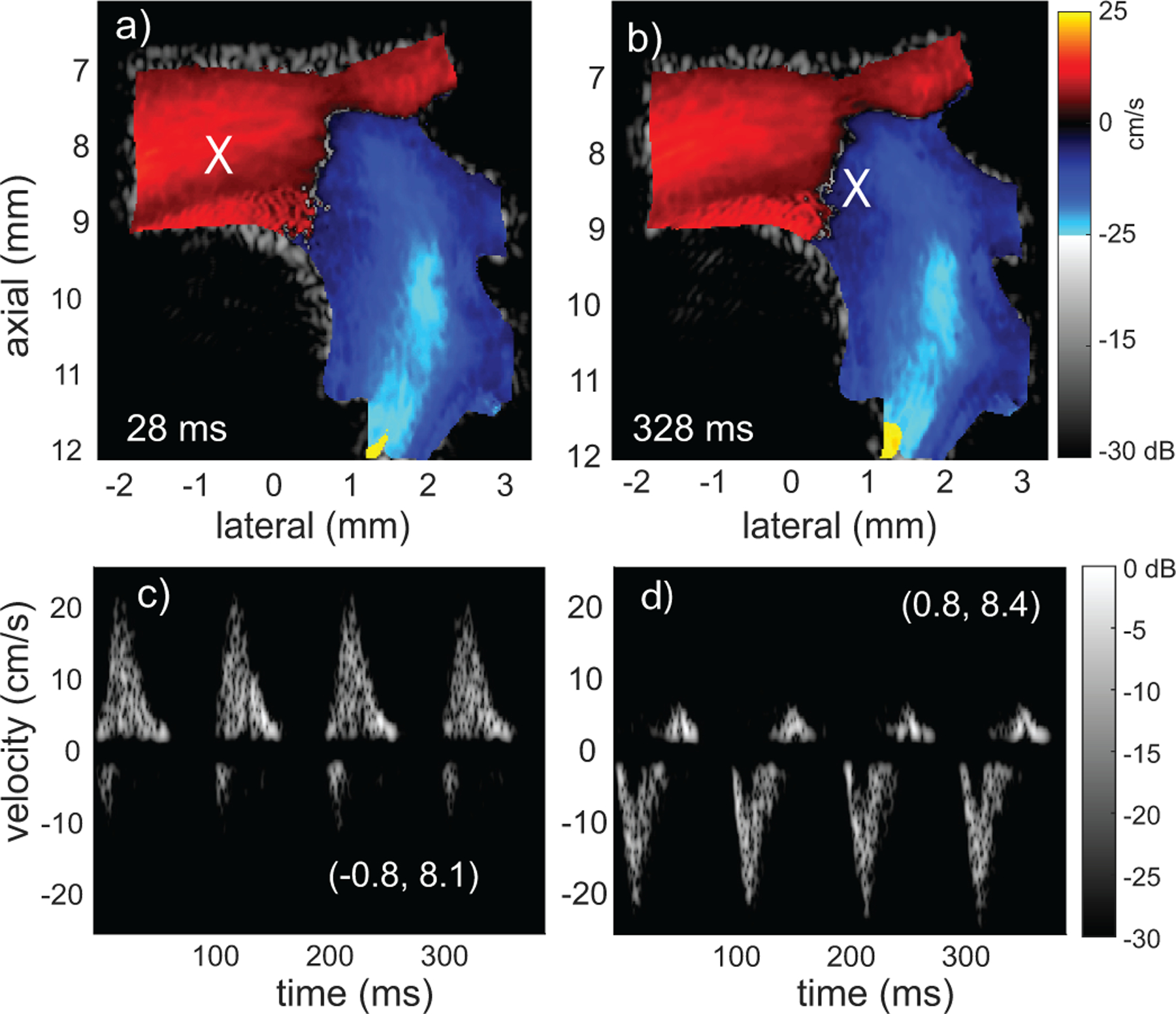
Color Doppler and spectrogram examples from a 4 cardiac cycle simulation with a 1-mm/s velocity threshold, distance threshold of 2*d, $0.25\;pts{\text{/λ}}^{\text{3}}$) seed concentration and 10-kHz effective PRF. Color Doppler frames for matching phases of the cardiac cycle at a) 28 ms and b) 328 ms. Spectrograms acquired at X markings of (a,b) with dominant upward flow [−0.8, 8.1] mm and b) dominant downward flow [0.8, 8.4] mm. The movie sequence (Mm3) reveals aliasing from ≈ 6–29 ms and highlights the repetitive nature of the flow.

Color Doppler examples are shown at 28 and 328 ms [Figure 9(a) and (b)]. The images were effectively identical with some subtle variation as would be observed *in vivo*. The time points are just after the peak flow and a small patch of aliasing is visible at the exit of the descending aorta.Doppler waveforms [Figs. 9(c) and (d)] at the points [−0.8, 8.1] and [0.8, 8.4] mm are marked with an X in Figs. 9(a) and (b). The spectrograms showed consistent features from cycle to cycle with the slight variation that would be observed *in vivo*. Flow reversal was observed at each location and the peak flow occurred near 17 ms.

**FIGURE 10.**
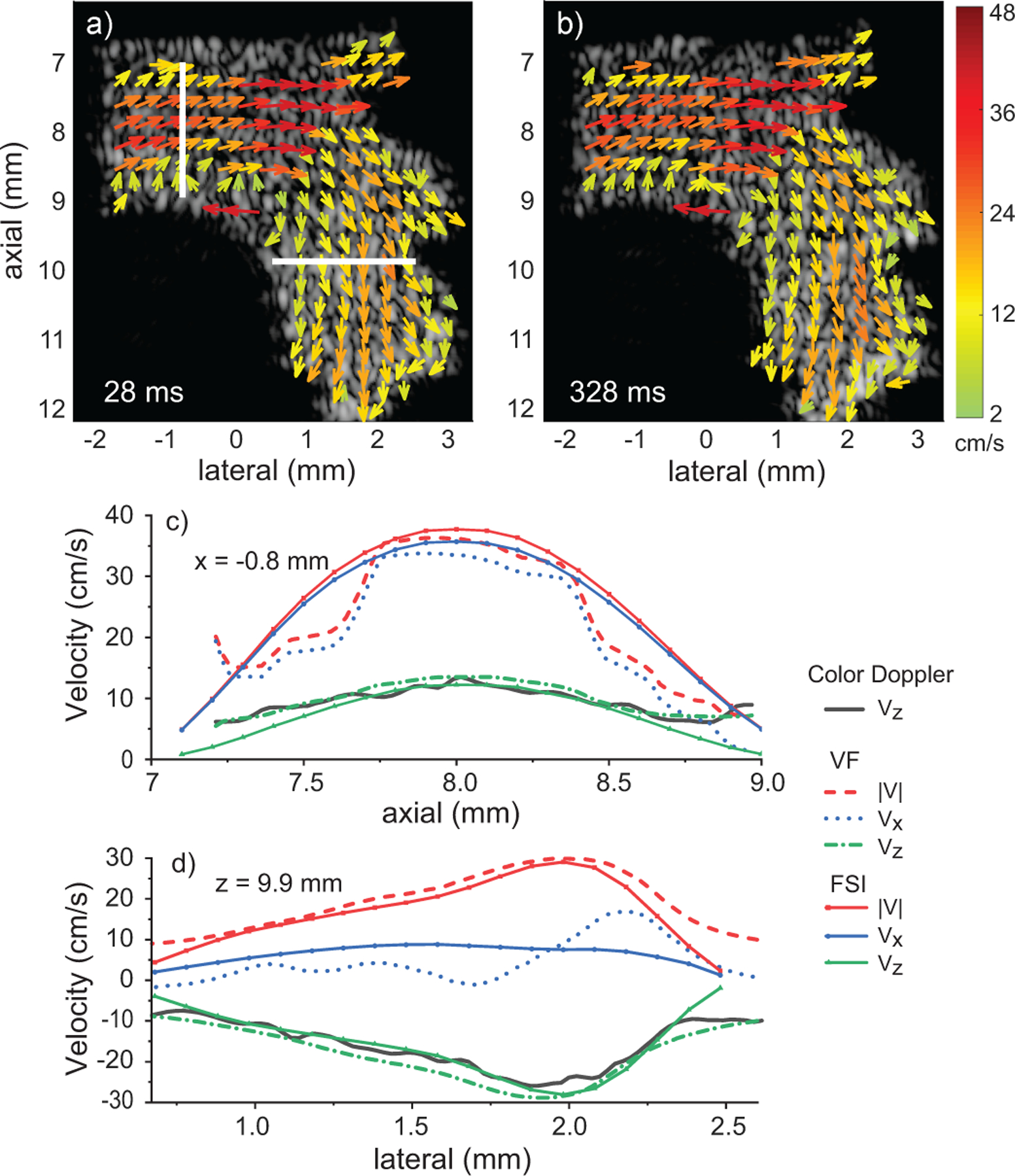
Ultrasound vector-flow (VF) estimates over 4 cardiac cycles. Vector patterning was consistent at the same time point in cardiac cycle: a) 28 and b) 328 ms. The flow profiles of the FSI, vector flow and color Doppler are plotted for the slices at c) x=−0.8 and d) z=9.9mm (marked with white lines). The movie sequence (Mm4) reveals the repetitive features of vector flow including back flow.

Vector-flow estimates at peak diastole flow at 28 and 328 ms [Figs. 10(a) and (b), respectively, and Mm4] are also nearly identical. The transition zone between red and blue in the color Doppler [Figure 9(a) and (b)] is revealed to be horizontal flow. The vector-flow and color Doppler at the slices marked with white lines are compared to the FSI values at the same slice location [Figs. 10(c), (d)]. For the x=−0.8mm slice, the vector estimates and FSI values agreed to within ≈ 10% across the whole span for $V_{z}$ and in the central region for $V_{x}$ and $\left|V\right|$. For the z=9.9mm slice, the vector estimates and FSI values agreed to within ≈ 10% across the whole span for $V_{z}$ and $\left|V\right|$ but the bias was generally large across the whole span for $V_{x}$. The overall results are consistent with an increased estimator error as the flow becomes more horizontal.

**FIGURE 11.**
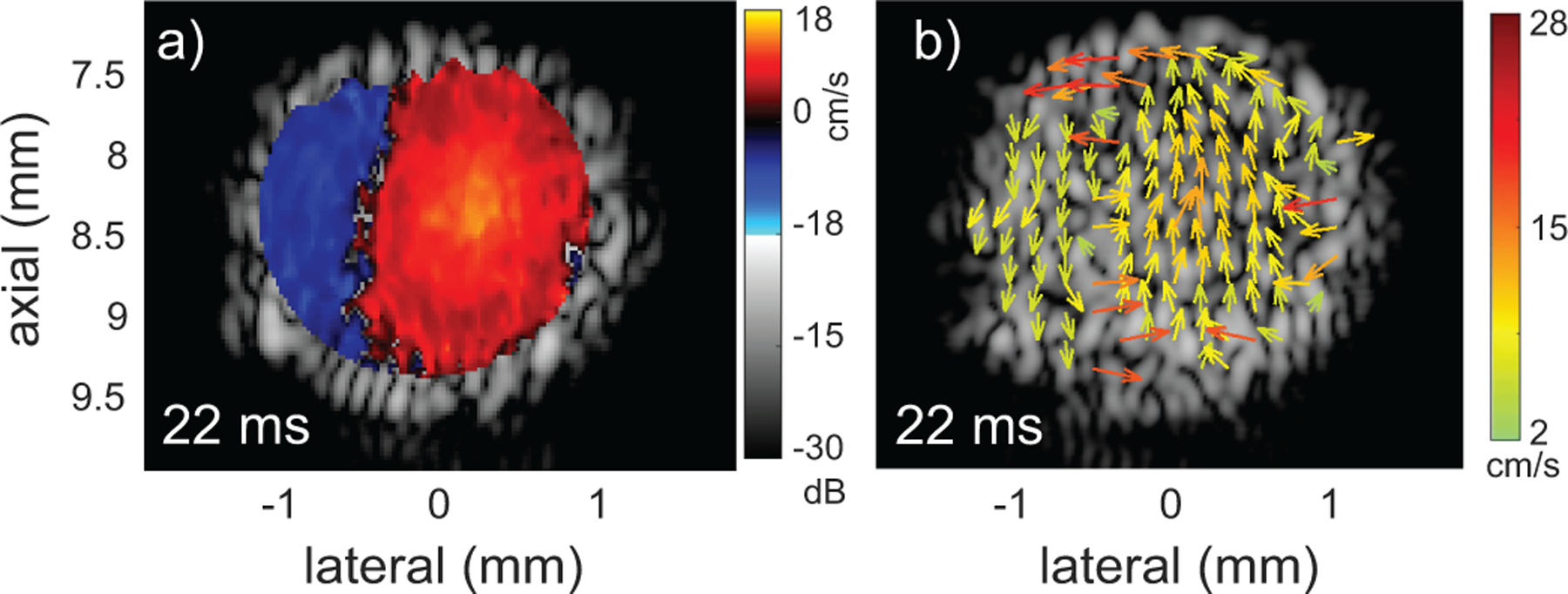
a) Color Doppler and b) vector-flow simulation in the x=0 plane of Figure 2. The upward and downward flow in the color Doppler image is revealed to be rotation in the vector-flow image. Because flow is primarily through the image plane, the rotation is indicative of forward helical flow.

The tracking of the 3D flow field allows imaging in an arbitrary image plane. For instance, Figure 11 show a simulation in the cross section of the ascending aorta at the x=0 plane of Figure 2. The simulation conditions are the same as above in the Y=0mm plane and the dominant flow direction is through the image plane. During the systolic phase (≈ 22 ms) the color Doppler shows flow in an upward and downward direction. In the vector flow image [Figure 2(b)], the flow pattern is revealed to have a counter-clockwise rotational component which is indicative of forward helical flow. Helical flow in the aorta is observed in human subjects [18], [19] and also in mice [20].

## IV. Discussion

One issue with a flow simulation in mice or humans is whether the output truly recreates what would be observed with ultrasound, MRI, etc. Our scatterer tracking approach addresses the problem of validating an FDM by allowing us to “close the loop” meaning that we could use ultrasound measurements to inform the FDM forcing function, such as in [21]–[23], generate a fluid domain model, generate a scatterer model, derive an ultrasound simulation and then compare the simulation to the original input measurements, ideally using *in vivo* vector-flow measurements to derive the forcing function. Our approach allows any discrepancies between ultrasound input and ultrasound simulation to be identified which would then allow for refinement of the FDM.

We chose the Carreau–Yasuda model to represent the apparent viscosity of blood because it effectively captures the shear-thinning behavior that arises from red blood cell (RBC) dynamics. As RBCs deform, align, and aggregate in response to varying shear conditions, they induce non-Newtonian flow characteristics that must be accounted for in continuum models. Unlike simpler models such as the power-law or Casson models, the Carreau–Yasuda formulation offers greater flexibility by incorporating the zero-shear and infinite-shear viscosity limits, along with parameters that control the smoothness of the transition across physiological shear rates. This makes it particularly well-suited for vascular simulations where local shear rates span several orders of magnitude. Importantly, the Carreau–Yasuda model avoids nonphysical behavior at very low or high shear rates and has been widely validated against *in vitro* measurements of whole blood viscosity. Thus, it provides a robust and physiologically informed means to capture the effective macroscopic consequences of microscopic RBC dynamics without explicitly resolving individual cells.

The choice of ultrasound simulator and post-processing approach is not the focus of our method and any simulation tool using a set of points as input will yield similar synthetic ultrasound data. As in [24], we did not inject noise into the simulated signal because our focus was on the method of continuous particle tracking rather then optimizing post processing methods. A comparison of simulated vector flow to *in vivo* vector flow measurements would require noise in the signal to match the measurement system noise. Using the Verasonics simulation engine, we were able to generate raw data (no beamforming) for 320 plane-wave transmits in one minute whereas, for reference, Field II [25] required 47 seconds to generate raw data for a single transmit event. Therefore, the time to generate the raw channel data for each time step is dominated by the ultrasound simulator not the particle tracking.

We tracked scatterers in the full flow volume because the fluid motion is truly in 3D [Figure 3] and *in vivo* 2D ultrasound measurements are influenced by the 3D motion path. With our approach, scatterers can enter or exit the image plane just as would occur *in vivo*. Simplifying the simulation by removing the y displacement would no longer replicate the *in vivo* flow. The ability to simulate or to acquire data in an arbitrary slice plane offers additional opportunities to refine an FDM model by comparing to *in vivo* ultrasound measurements and to locate unique characteristics of disease models such as abnormal helical flow patterns.

Refresh zones should be placed at any port where fluid can enter or exit the simulation. For our aorta example, removing the refresh zones at the carotid branches and descending aorta would result in voids entering the simulation because of the backflow that occurred each cardiac cycle. After selecting the refresh zones, the local concentration should be tracked over time to ensure it is stable. The simulation PRF will impact the zone selection because the lower the PRF, the further the scatterers move each time step. Once a refresh zone has been established for some PRF, a simple way to manage cases with a decreased PRF without changing the refresh zones is to implement multiple intermediate time steps at a higher PRF before passing the scatterers to the ultrasound simulator. As noted in Figure 4(b), it is important to randomly repopulate the refresh zones each time step or the scatterer flow lines will become identical each cardiac cycle.

Ultrasound simulations of complex flow offer several practical uses such as evaluating expected *in vivo* performance of probes, implementing and optimizing new transmit schemes, and developing algorithms to quantify features of complex flow. For instance, because we track the full scatterer volume, we could simulate volumetric acquisition with a high-frequency row-column array under realistic *in vivo* flow conditions or we could implement anti-aliasing approaches, such as a staggered-PRF [26], into our pipeline. Aliasing is of particular concern for small-animal imaging because the increased center frequency inherently lowers the Doppler aliasing limit.

## V. Conclusion

We presented a technique to continuously track, without the need for random reseeding, a collection of particles over an arbitrary number of cardiac cycles using an FDM to define the flow field velocity history. The particle collection at each time step can be passed to any ultrasound simulator that uses point targets to model tissue. Our approach is of primary use for cases of complex, pulsatile *in vivo* blood flow where reverse, rotational, out-of-plane and/or helical flow may exist. Such flow requires careful management of the scatterers to avoid large swings in local particle concentration.

Our particle tracking approach allows for the replication of *in vivo*, high-speed plane-wave acquisitions with no slow-time discontinuities that arise when particles are periodically reseeded to maintain concentration. Using a prior FSI model of a mouse aorta [9], we demonstrated the process of preparing an FDM, generating and tracking a scatterer collection, creating a synthetic high-frequency, high-speed plane-wave simulation with a 15-MHz linear array, and post processing the data to produce color Doppler and vector flow estimates. Our approach helps “close the loop” between *in vivo* ultrasound measurement and FDM simulations and opens the door to using vector-flow measurements to improve accuracy of FDM models of complex flow.

## Supplementary Material

Mm1

Mm4

Mm3

Mm2

## Figures and Tables

**TABLE 1.** FSI Related Parameters

Parameter	Value
Blood Density	1027 kg/m^3^
$\eta_{\infty }$	2 cP
$\eta_{0}$	11 cP
n	0.71
a	0.2
λ	1.5
Mesh Points/Volume	147,538
Volumes	51
Time Step	2 ms
Cardiac Cycle	100 ms
Interpolation Grid Spacing (*d*)	0.1 mm
Grid Flow Points/Volume	27,937

**TABLE 2.** Scatterer concentration time history descriptive statistics as a function of velocity (V) and distance (D) thresholds.

V Thresh	$\text{D}\;\text{Thresh}\left(\text{n*}d\right)$	Mean	SD	Int	Mean-Int

mm/s	n	${\text{pts/λ}}^{\text{3}}$	${\text{pts/λ}}^{\text{3}}$	${\text{pts/λ}}^{\text{3}}$	${\text{pts/λ}}^{\text{3}}$
0	2	0.341	0.035	0.294	0.047
0.1	2	0.304	0.025	0.287	0.017
1	2	0.278	0.022	0.278	−0.001
1	5	0.279	0.026	0.280	0.000
1	15	0.278	0.023	0.284	−0.006
2	2	0.256	0.022	0.253	0.004
10	2	0.210	0.032	0.228	−0.017
10	3	0.194	0.034	0.217	−0.023
10	15	0.166	0.056	0.191	−0.026

SD = Standard deviation; Int = Linear fit intercept. A Mean-Intercept value of zero indicates a stable concentration.
